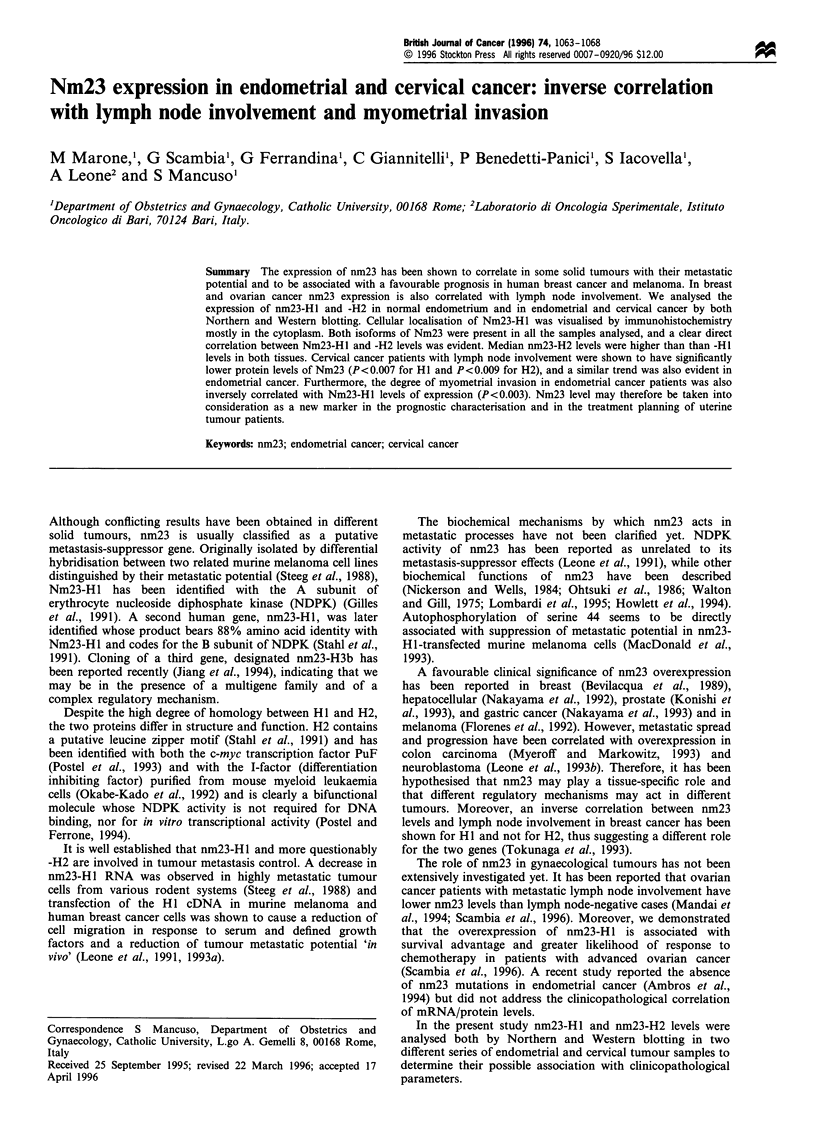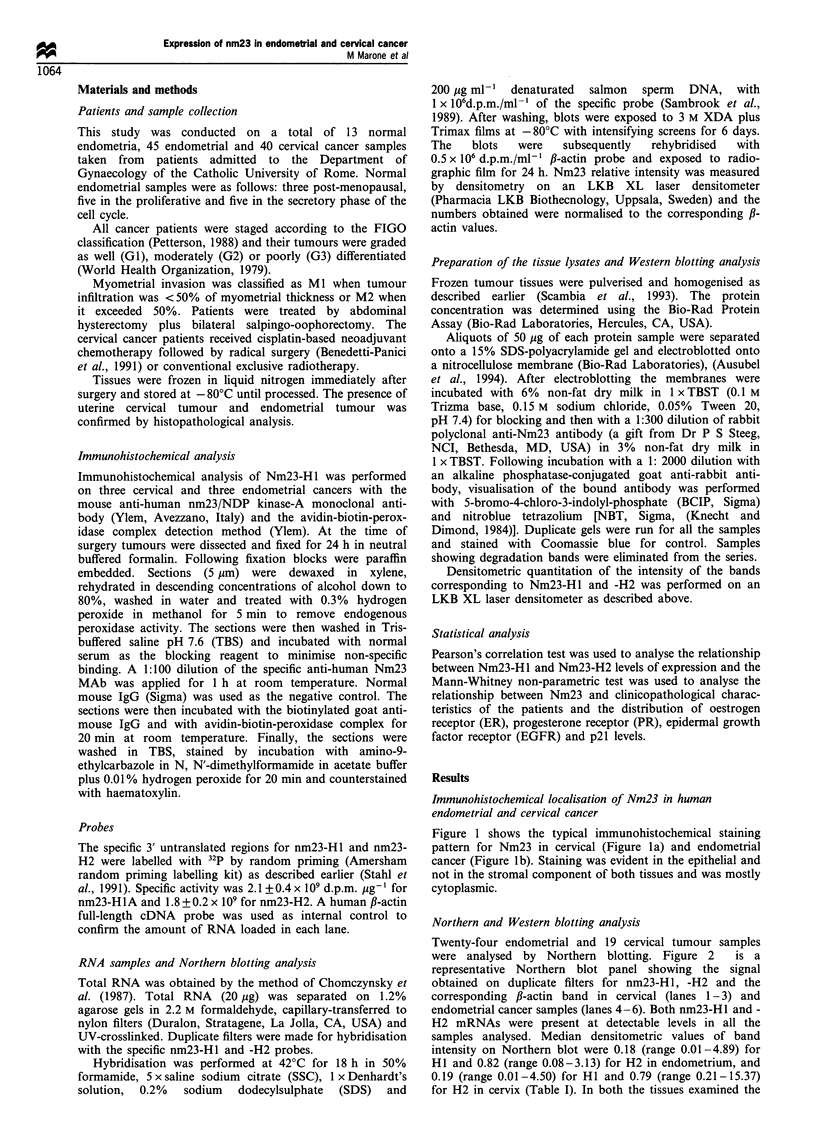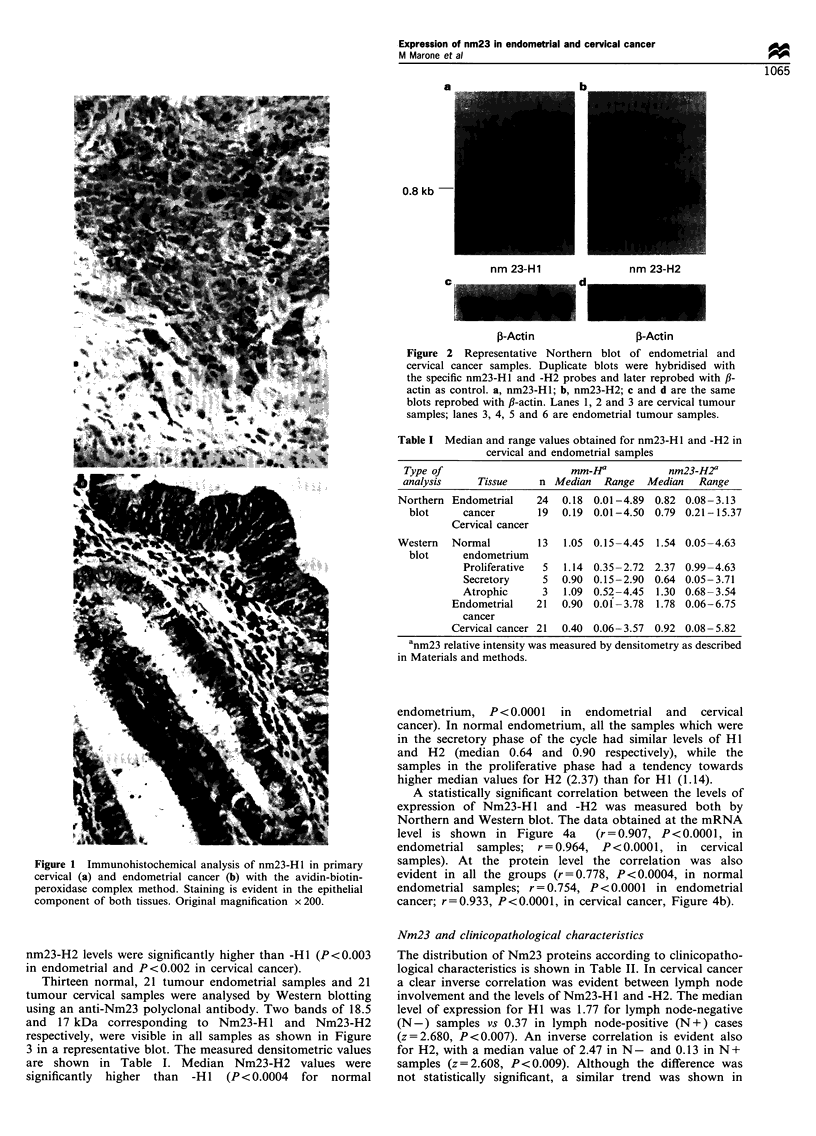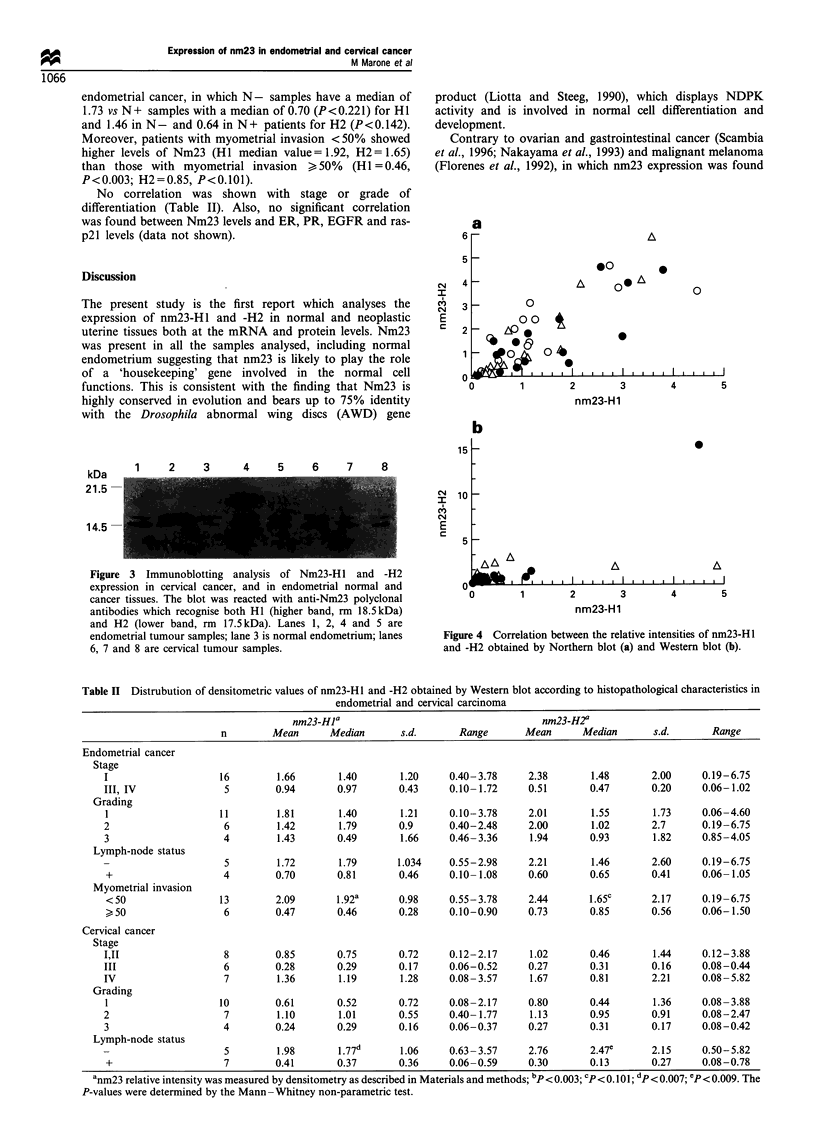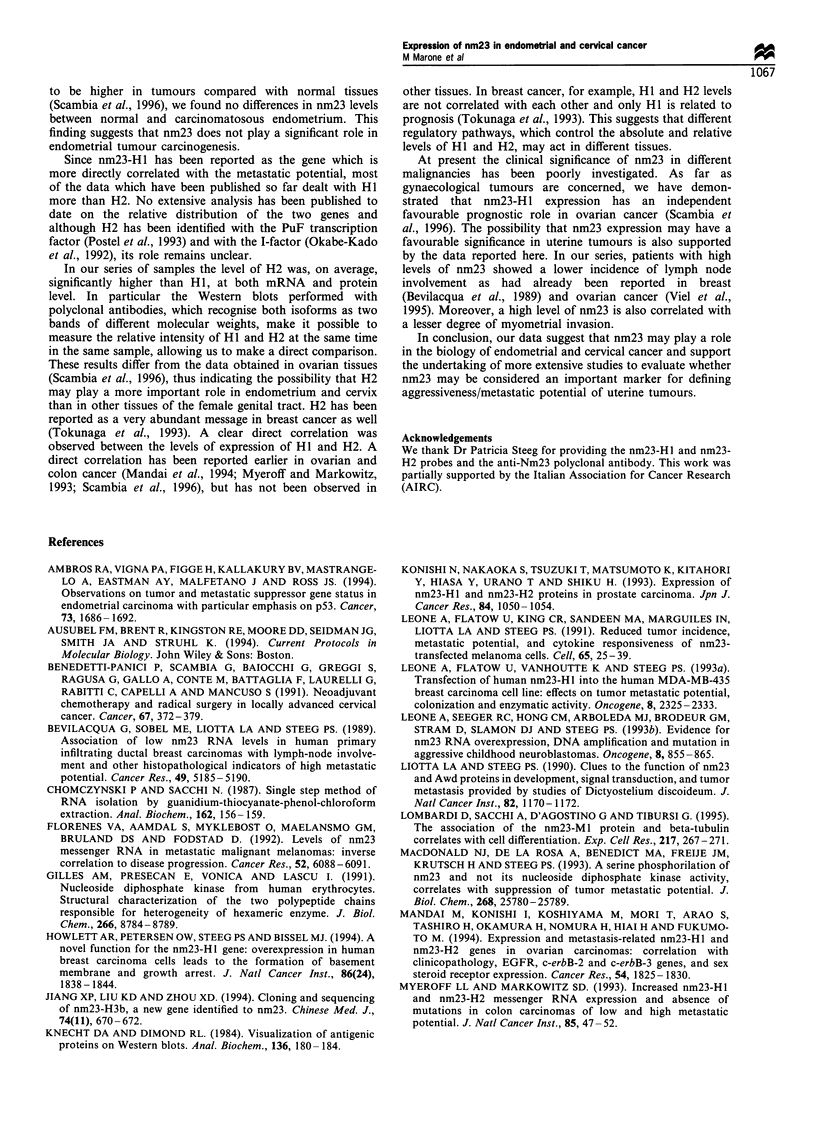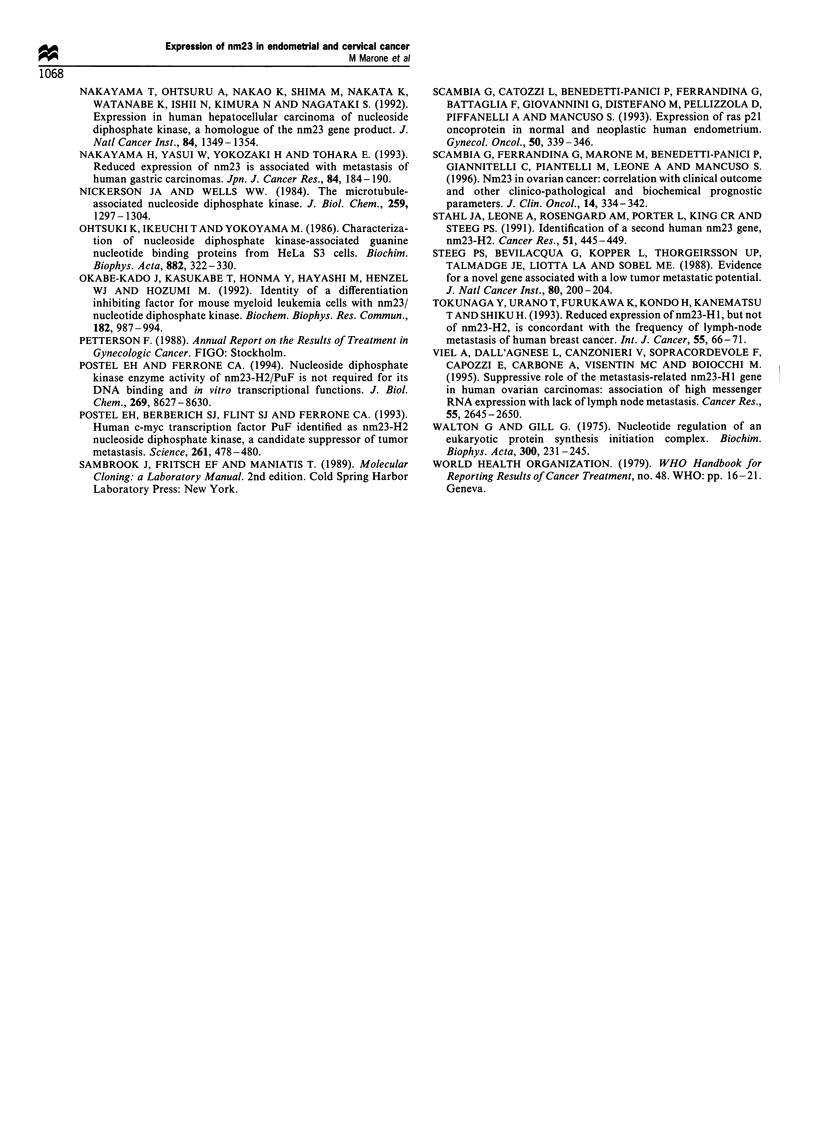# Nm23 expression in endometrial and cervical cancer: inverse correlation with lymph node involvement and myometrial invasion.

**DOI:** 10.1038/bjc.1996.490

**Published:** 1996-10

**Authors:** M. Marone, G. Scambia, G. Ferrandina, C. Giannitelli, P. Benedetti-Panici, S. Iacovella, A. Leone, S. Mancuso

**Affiliations:** Department of Obstetrics and Gynaecology, Catholic University, Rome, Italy.

## Abstract

**Images:**


British Journal of Cancer (1996) 74, 1063-1068
© 1996 Stockton Press All rights reserved 0007-0920/96 $12.00

### Nm23 expression in endometrial and cervical cancer: inverse correlation with lymph node involvement and myometrial invasion

M Marone,[1], G Scambia[1], G Ferrandina[1], C Giannitelli[1], P Benedetti-Panici[1], S Iacovella[1], A Leone[2] and S Mancuso[1]

[1]*Department of Obstetrics and Gynaecology, Catholic University, 00168 Rome;* [2]*Laboratorio di Oncologia Sperimentale, Istituto Oncologico di Bari, 70124 Bari, Italy.*

**Summary** The expression of nm23 has been shown to correlate in some solid tumours with their metastatic potential and to be associated with a favourable prognosis in human breast cancer and melanoma. In breast and ovarian cancer nm23 expression is also correlated with lymph node involvement. We analysed the expression of nm23-H1 and -H2 in normal endometrium and in endometrial and cervical cancer by both Northern and Western blotting. Cellular localisation of Nm23-H1 was visualised by immunohistochemistry mostly in the cytoplasm. Both isoforms of Nm23 were present in all the samples analysed, and a clear direct correlation between Nm23-H1 and -H2 levels was evident. Median nm23-H2 levels were higher than than -H1 levels in both tissues. Cervical cancer patients with lymph node involvement were shown to have significantly lower protein levels of Nm23 ($P<0.007$ for H1 and $P<0.009$ for H2), and a similar trend was also evident in endometrial cancer. Furthermore, the degree of myometrial invasion in endometrial cancer patients was also inversely correlated with Nm23-H1 levels of expression ($P<0.003$). Nm23 level may therefore be taken into consideration as a new marker in the prognostic characterisation and in the treatment planning of uterine tumour patients.

**Keywords:** nm23; endometrial cancer; cervical cancer

Although conflicting results have been obtained in different solid tumours, nm23 is usually classified as a putative metastasis-suppressor gene. Originally isolated by differential hybridisation between two related murine melanoma cell lines distinguished by their metastatic potential (Steeg *et al.*, 1988), Nm23-H1 has been identified with the A subunit of erythrocyte nucleoside diphosphate kinase (NDPK) (Gilles *et al.*, 1991). A second human gene, nm23-H1, was later identified whose product bears 88% amino acid identity with Nm23-H1 and codes for the B subunit of NDPK (Stahl *et al.*, 1991). Cloning of a third gene, designated nm23-H3b has been reported recently (Jiang *et al.*, 1994), indicating that we may be in the presence of a multigene family and of a complex regulatory mechanism.

Despite the high degree of homology between H1 and H2, the two proteins differ in structure and function. H2 contains a putative leucine zipper motif (Stahl *et al.*, 1991) and has been identified with both the c-*myc* transcription factor PuF (Postel *et al.*, 1993) and with the I-factor (differentiation inhibiting factor) purified from mouse myeloid leukaemia cells (Okabe-Kado *et al.*, 1992) and is clearly a bifunctional molecule whose NDPK activity is not required for DNA binding, nor for *in vitro* transcriptional activity (Postel and Ferrone, 1994).

It is well established that nm23-H1 and more questionably -H2 are involved in tumour metastasis control. A decrease in nm23-H1 RNA was observed in highly metastatic tumour cells from various rodent systems (Steeg *et al.*, 1988) and transfection of the H1 cDNA in murine melanoma and human breast cancer cells was shown to cause a reduction of cell migration in response to serum and defined growth factors and a reduction of tumour metastatic potential '*in vivo*' (Leone *et al.*, 1991, 1993*a*).

The biochemical mechanisms by which nm23 acts in metastatic processes have not been clarified yet. NDPK activity of nm23 has been reported as unrelated to its metastasis-suppressor effects (Leone *et al.*, 1991), while other biochemical functions of nm23 have been described (Nickerson and Wells, 1984; Ohtsuki *et al.*, 1986; Walton and Gill, 1975; Lombardi *et al.*, 1995; Howlett *et al.*, 1994). Autophosphorylation of serine 44 seems to be directly associated with suppression of metastatic potential in nm23-H1-transfected murine melanoma cells (MacDonald *et al.*, 1993).

A favourable clinical significance of nm23 overexpression has been reported in breast (Bevilacqua *et al.*, 1989), hepatocellular (Nakayama *et al.*, 1992), prostate (Konishi *et al.*, 1993), and gastric cancer (Nakayama *et al.*, 1993) and in melanoma (Florenes *et al.*, 1992). However, metastatic spread and progression have been correlated with overexpression in colon carcinoma (Myeroff and Markowitz, 1993) and neuroblastoma (Leone *et al.*, 1993*b*). Therefore, it has been hypothesised that nm23 may play a tissue-specific role and that different regulatory mechanisms may act in different tumours. Moreover, an inverse correlation between nm23 levels and lymph node involvement in breast cancer has been shown for H1 and not for H2, thus suggesting a different role for the two genes (Tokunaga *et al.*, 1993).

The role of nm23 in gynaecological tumours has not been extensively investigated yet. It has been reported that ovarian cancer patients with metastatic lymph node involvement have lower nm23 levels than lymph node-negative cases (Mandai *et al.*, 1994; Scambia *et al.*, 1996). Moreover, we demonstrated that the overexpression of nm23-H1 is associated with survival advantage and greater likelihood of response to chemotherapy in patients with advanced ovarian cancer (Scambia *et al.*, 1996). A recent study reported the absence of nm23 mutations in endometrial cancer (Ambros *et al.*, 1994) but did not address the clinicopathological correlation of mRNA/protein levels.

In the present study nm23-H1 and nm23-H2 levels were analysed both by Northern and Western blotting in two different series of endometrial and cervical tumour samples to determine their possible association with clinicopathological parameters.

Correspondence S Mancuso, Department of Obstetrics and Gynaecology, Catholic University, L.go A. Gemelli 8, 00168 Rome, Italy
Received 25 September 1995; revised 22 March 1996; accepted 17 April 1996

### Materials and methods

*Patients and sample collection*

This study was conducted on a total of 13 normal endometria, 45 endometrial and 40 cervical cancer samples taken from patients admitted to the Department of Gynaecology of the Catholic University of Rome. Normal endometrial samples were as follows: three post-menopausal, five in the proliferative and five in the secretory phase of the cell cycle.

All cancer patients were staged according to the FIGO classification (Petterson, 1988) and their tumours were graded as well (G1), moderately (G2) or poorly (G3) differentiated (World Health Organization, 1979).

Myometrial invasion was classified as M1 when tumour infiltration was <50% of myometrial thickness or M2 when it exceeded 50%. Patients were treated by abdominal hysterectomy plus bilateral salpingo-oophorectomy. The cervical cancer patients received cisplatin-based neoadjuvant chemotherapy followed by radical surgery (Benedetti-Panici *et al.*, 1991) or conventional exclusive radiotherapy.

Tissues were frozen in liquid nitrogen immediately after surgery and stored at −80°C until processed. The presence of uterine cervical tumour and endometrial tumour was confirmed by histopathological analysis.

*Immunohistochemical analysis*

Immunohistochemical analysis of Nm23-H1 was performed on three cervical and three endometrial cancers with the mouse anti-human nm23/NDP kinase-A monoclonal antibody (Ylem, Avezzano, Italy) and the avidin-biotin-peroxidase complex detection method (Ylem). At the time of surgery tumours were dissected and fixed for 24 h in neutral buffered formalin. Following fixation blocks were paraffin embedded. Sections (5 μm) were dewaxed in xylene, rehydrated in descending concentrations of alcohol down to 80%, washed in water and treated with 0.3% hydrogen peroxide in methanol for 5 min to remove endogenous peroxidase activity. The sections were then washed in Tris-buffered saline pH 7.6 (TBS) and incubated with normal serum as the blocking reagent to minimise non-specific binding. A 1:100 dilution of the specific anti-human Nm23 MAb was applied for 1 h at room temperature. Normal mouse IgG (Sigma) was used as the negative control. The sections were then incubated with the biotinylated goat anti-mouse IgG and with avidin-biotin-peroxidase complex for 20 min at room temperature. Finally, the sections were washed in TBS, stained by incubation with amino-9-ethylcarbazole in N, N′-dimethylformamide in acetate buffer plus 0.01% hydrogen peroxide for 20 min and counterstained with haematoxylin.

*Probes*

The specific 3′ untranslated regions for nm23-H1 and nm23-H2 were labelled with $^{32}P$ by random priming (Amersham random priming labelling kit) as described earlier (Stahl *et al.*, 1991). Specific activity was $2.1 \pm 0.4 \times 10^9$ d.p.m. $\mu g^{-1}$ for nm23-H1A and $1.8 \pm 0.2 \times 10^9$ for nm23-H2. A human β-actin full-length cDNA probe was used as internal control to confirm the amount of RNA loaded in each lane.

*RNA samples and Northern blotting analysis*

Total RNA was obtained by the method of Chomczynsky *et al.* (1987). Total RNA (20 μg) was separated on 1.2% agarose gels in 2.2 M formaldehyde, capillary-transferred to nylon filters (Duralon, Stratagene, La Jolla, CA, USA) and UV-crosslinked. Duplicate filters were made for hybridisation with the specific nm23-H1 and -H2 probes.

Hybridisation was performed at 42°C for 18 h in 50% formamide, 5 × saline sodium citrate (SSC), 1 × Denhardt's solution, 0.2% sodium dodecylsulphate (SDS) and 200 $\mu g\ ml^{-1}$ denaturated salmon sperm DNA, with $1 \times 10^6$d.p.m./$ml^{-1}$ of the specific probe (Sambrook *et al.*, 1989). After washing, blots were exposed to 3 M XDA plus Trimax films at −80°C with intensifying screens for 6 days. The blots were subsequently rehybridised with $0.5 \times 10^6$ d.p.m./$ml^{-1}$ β-actin probe and exposed to radiographic film for 24 h. Nm23 relative intensity was measured by densitometry on an LKB XL laser densitometer (Pharmacia LKB Biothecnology, Uppsala, Sweden) and the numbers obtained were normalised to the corresponding β-actin values.

*Preparation of the tissue lysates and Western blotting analysis*

Frozen tumour tissues were pulverised and homogenised as described earlier (Scambia *et al.*, 1993). The protein concentration was determined using the Bio-Rad Protein Assay (Bio-Rad Laboratories, Hercules, CA, USA).

Aliquots of 50 μg of each protein sample were separated onto a 15% SDS-polyacrylamide gel and electroblotted onto a nitrocellulose membrane (Bio-Rad Laboratories), (Ausubel *et al.*, 1994). After electroblotting the membranes were incubated with 6% non-fat dry milk in 1 × TBST (0.1 M Trizma base, 0.15 M sodium chloride, 0.05% Tween 20, pH 7.4) for blocking and then with a 1:300 dilution of rabbit polyclonal anti-Nm23 antibody (a gift from Dr P S Steeg, NCI, Bethesda, MD, USA) in 3% non-fat dry milk in 1 × TBST. Following incubation with a 1: 2000 dilution with an alkaline phosphatase-conjugated goat anti-rabbit antibody, visualisation of the bound antibody was performed with 5-bromo-4-chloro-3-indolyl-phosphate (BCIP, Sigma) and nitroblue tetrazolium [NBT, Sigma, (Knecht and Dimond, 1984)]. Duplicate gels were run for all the samples and stained with Coomassie blue for control. Samples showing degradation bands were eliminated from the series.

Densitometric quantitation of the intensity of the bands corresponding to Nm23-H1 and -H2 was performed on an LKB XL laser densitometer as described above.

*Statistical analysis*

Pearson's correlation test was used to analyse the relationship between Nm23-H1 and Nm23-H2 levels of expression and the Mann-Whitney non-parametric test was used to analyse the relationship between Nm23 and clinicopathological characteristics of the patients and the distribution of oestrogen receptor (ER), progesterone receptor (PR), epidermal growth factor receptor (EGFR) and p21 levels.

### Results

*Immunohistochemical localisation of Nm23 in human endometrial and cervical cancer*

Figure 1 shows the typical immunohistochemical staining pattern for Nm23 in cervical (Figure 1a) and endometrial cancer (Figure 1b). Staining was evident in the epithelial and not in the stromal component of both tissues and was mostly cytoplasmic.

*Northern and Western blotting analysis*

Twenty-four endometrial and 19 cervical tumour samples were analysed by Northern blotting. Figure 2 is a representative Northern blot panel showing the signal obtained on duplicate filters for nm23-H1, -H2 and the corresponding β-actin band in cervical (lanes 1–3) and endometrial cancer samples (lanes 4–6). Both nm23-H1 and -H2 mRNAs were present at detectable levels in all the samples analysed. Median densitometric values of band intensity on Northern blot were 0.18 (range 0.01–4.89) for H1 and 0.82 (range 0.08–3.13) for H2 in endometrium, and 0.19 (range 0.01–4.50) for H1 and 0.79 (range 0.21–15.37) for H2 in cervix (Table I). In both the tissues examined the

**Figure 1** Immunohistochemical analysis of nm23-H1 in primary cervical (**a**) and endometrial cancer (**b**) with the avidin-biotin-peroxidase complex method. Staining is evident in the epithelial component of both tissues. Original magnification ×200.

nm23-H2 levels were significantly higher than -H1 ($P<0.003$ in endometrial and $P<0.002$ in cervical cancer).

Thirteen normal, 21 tumour endometrial samples and 21 tumour cervical samples were analysed by Western blotting using an anti-Nm23 polyclonal antibody. Two bands of 18.5 and 17 kDa corresponding to Nm23-H1 and Nm23-H2 respectively, were visible in all samples as shown in Figure 3 in a representative blot. The measured densitometric values are shown in Table I. Median Nm23-H2 values were significantly higher than -H1 ($P<0.0004$ for normal

**Figure 2** Representative Northern blot of endometrial and cervical cancer samples. Duplicate blots were hybridised with the specific nm23-H1 and -H2 probes and later reprobed with β-actin as control. **a**, nm23-H1; **b**, nm23-H2; **c** and **d** are the same blots reprobed with β-actin. Lanes 1, 2 and 3 are cervical tumour samples; lanes 3, 4, 5 and 6 are endometrial tumour samples.

**Table I** Median and range values obtained for nm23-H1 and -H2 in cervical and endometrial samples

| *Type of analysis* | *Tissue* | n | *mm-H[a]* *Median* | *Range* | *nm23-H2[a]* *Median* | *Range* |
|---|---|---|---|---|---|---|
| Northern blot | Endometrial cancer | 24 | 0.18 | 0.01–4.89 | 0.82 | 0.08–3.13 |
| | Cervical cancer | 19 | 0.19 | 0.01–4.50 | 0.79 | 0.21–15.37 |
| Western blot | Normal endometrium | 13 | 1.05 | 0.15–4.45 | 1.54 | 0.05–4.63 |
| | Proliferative | 5 | 1.14 | 0.35–2.72 | 2.37 | 0.99–4.63 |
| | Secretory | 5 | 0.90 | 0.15–2.90 | 0.64 | 0.05–3.71 |
| | Atrophic | 3 | 1.09 | 0.52–4.45 | 1.30 | 0.68–3.54 |
| | Endometrial cancer | 21 | 0.90 | 0.01–3.78 | 1.78 | 0.06–6.75 |
| | Cervical cancer | 21 | 0.40 | 0.06–3.57 | 0.92 | 0.08–5.82 |

[a]nm23 relative intensity was measured by densitometry as described in Materials and methods.

endometrium, $P<0.0001$ in endometrial and cervical cancer). In normal endometrium, all the samples which were in the secretory phase of the cycle had similar levels of H1 and H2 (median 0.64 and 0.90 respectively), while the samples in the proliferative phase had a tendency towards higher median values for H2 (2.37) than for H1 (1.14).

A statistically significant correlation between the levels of expression of Nm23-H1 and -H2 was measured both by Northern and Western blot. The data obtained at the mRNA level is shown in Figure 4a ($r=0.907$, $P<0.0001$, in endometrial samples; $r=0.964$, $P<0.0001$, in cervical samples). At the protein level the correlation was also evident in all the groups ($r=0.778$, $P<0.0004$, in normal endometrial samples; $r=0.754$, $P<0.0001$ in endometrial cancer; $r=0.933$, $P<0.0001$, in cervical cancer, Figure 4b).

### *Nm23 and clinicopathological characteristics*

The distribution of Nm23 proteins according to clinicopathological characteristics is shown in Table II. In cervical cancer a clear inverse correlation was evident between lymph node involvement and the levels of Nm23-H1 and -H2. The median level of expression for H1 was 1.77 for lymph node-negative (N−) samples *vs* 0.37 in lymph node-positive (N+) cases ($z=2.680$, $P<0.007$). An inverse correlation is evident also for H2, with a median value of 2.47 in N− and 0.13 in N+ samples ($z=2.608$, $P<0.009$). Although the difference was not statistically significant, a similar trend was shown in endometrial cancer, in which N− samples have a median of 1.73 *vs* N+ samples with a median of 0.70 ($P<0.221$) for H1 and 1.46 in N− and 0.64 in N+ patients for H2 ($P<0.142$). Moreover, patients with myometrial invasion <50% showed higher levels of Nm23 (H1 median value = 1.92, H2 = 1.65) than those with myometrial invasion ⩾50% (H1 = 0.46, $P<0.003$; H2 = 0.85, $P<0.101$).

No correlation was shown with stage or grade of differentiation (Table II). Also, no significant correlation was found between Nm23 levels and ER, PR, EGFR and ras-p21 levels (data not shown).

### Discussion

The present study is the first report which analyses the expression of nm23-H1 and -H2 in normal and neoplastic uterine tissues both at the mRNA and protein levels. Nm23 was present in all the samples analysed, including normal endometrium suggesting that nm23 is likely to play the role of a 'housekeeping' gene involved in the normal cell functions. This is consistent with the finding that Nm23 is highly conserved in evolution and bears up to 75% identity with the *Drosophila* abnormal wing discs (AWD) gene product (Liotta and Steeg, 1990), which displays NDPK activity and is involved in normal cell differentiation and development.

Contrary to ovarian and gastrointestinal cancer (Scambia *et al.*, 1996; Nakayama *et al.*, 1993) and malignant melanoma (Florenes *et al.*, 1992), in which nm23 expression was found

**Figure 3** Immunoblotting analysis of Nm23-H1 and -H2 expression in cervical cancer, and in endometrial normal and cancer tissues. The blot was reacted with anti-Nm23 polyclonal antibodies which recognise both H1 (higher band, rm 18.5 kDa) and H2 (lower band, rm 17.5 kDa). Lanes 1, 2, 4 and 5 are endometrial tumour samples; lane 3 is normal endometrium; lanes 6, 7 and 8 are cervical tumour samples.

**Figure 4** Correlation between the relative intensities of nm23-H1 and -H2 obtained by Northern blot (**a**) and Western blot (**b**).

**Table II** Distrubution of densitometric values of nm23-H1 and -H2 obtained by Western blot according to histopathological characteristics in endometrial and cervical carcinoma

| | n | nm23-H1[a] Mean | Median | s.d. | Range | nm23-H2[a] Mean | Median | s.d. | Range |
|---|---|---|---|---|---|---|---|---|---|
| Endometrial cancer | | | | | | | | | |
| Stage | | | | | | | | | |
| I | 16 | 1.66 | 1.40 | 1.20 | 0.40–3.78 | 2.38 | 1.48 | 2.00 | 0.19–6.75 |
| III, IV | 5 | 0.94 | 0.97 | 0.43 | 0.10–1.72 | 0.51 | 0.47 | 0.20 | 0.06–1.02 |
| Grading | | | | | | | | | |
| 1 | 11 | 1.81 | 1.40 | 1.21 | 0.10–3.78 | 2.01 | 1.55 | 1.73 | 0.06–4.60 |
| 2 | 6 | 1.42 | 1.79 | 0.9 | 0.40–2.48 | 2.00 | 1.02 | 2.7 | 0.19–6.75 |
| 3 | 4 | 1.43 | 0.49 | 1.66 | 0.46–3.36 | 1.94 | 0.93 | 1.82 | 0.85–4.05 |
| Lymph-node status | | | | | | | | | |
| − | 5 | 1.72 | 1.79 | 1.034 | 0.55–2.98 | 2.21 | 1.46 | 2.60 | 0.19–6.75 |
| + | 4 | 0.70 | 0.81 | 0.46 | 0.10–1.08 | 0.60 | 0.65 | 0.41 | 0.06–1.05 |
| Myometrial invasion | | | | | | | | | |
| <50 | 13 | 2.09 | 1.92[a] | 0.98 | 0.55–3.78 | 2.44 | 1.65[c] | 2.17 | 0.19–6.75 |
| ⩾50 | 6 | 0.47 | 0.46 | 0.28 | 0.10–0.90 | 0.73 | 0.85 | 0.56 | 0.06–1.50 |
| Cervical cancer | | | | | | | | | |
| Stage | | | | | | | | | |
| I,II | 8 | 0.85 | 0.75 | 0.72 | 0.12–2.17 | 1.02 | 0.46 | 1.44 | 0.12–3.88 |
| III | 6 | 0.28 | 0.29 | 0.17 | 0.06–0.52 | 0.27 | 0.31 | 0.16 | 0.08–0.44 |
| IV | 7 | 1.36 | 1.19 | 1.28 | 0.08–3.57 | 1.67 | 0.81 | 2.21 | 0.08–5.82 |
| Grading | | | | | | | | | |
| 1 | 10 | 0.61 | 0.52 | 0.72 | 0.08–2.17 | 0.80 | 0.44 | 1.36 | 0.08–3.88 |
| 2 | 7 | 1.10 | 1.01 | 0.55 | 0.40–1.77 | 1.13 | 0.95 | 0.91 | 0.08–2.47 |
| 3 | 4 | 0.24 | 0.29 | 0.16 | 0.06–0.37 | 0.27 | 0.31 | 0.17 | 0.08–0.42 |
| Lymph-node status | | | | | | | | | |
| − | 5 | 1.98 | 1.77[d] | 1.06 | 0.63–3.57 | 2.76 | 2.47[e] | 2.15 | 0.50–5.82 |
| + | 7 | 0.41 | 0.37 | 0.36 | 0.06–0.59 | 0.30 | 0.13 | 0.27 | 0.08–0.78 |

[a]nm23 relative intensity was measured by densitometry as described in Materials and methods; [b]$P<0.003$; [c]$P<0.101$; [d]$P<0.007$; [e]$P<0.009$. The $P$-values were determined by the Mann–Whitney non-parametric test.

to be higher in tumours compared with normal tissues (Scambia *et al.*, 1996), we found no differences in nm23 levels between normal and carcinomatosous endometrium. This finding suggests that nm23 does not play a significant role in endometrial tumour carcinogenesis.

Since nm23-H1 has been reported as the gene which is more directly correlated with the metastatic potential, most of the data which have been published so far dealt with H1 more than H2. No extensive analysis has been published to date on the relative distribution of the two genes and although H2 has been identified with the PuF transcription factor (Postel *et al.*, 1993) and with the I-factor (Okabe-Kado *et al.*, 1992), its role remains unclear.

In our series of samples the level of H2 was, on average, significantly higher than H1, at both mRNA and protein level. In particular the Western blots performed with polyclonal antibodies, which recognise both isoforms as two bands of different molecular weights, make it possible to measure the relative intensity of H1 and H2 at the same time in the same sample, allowing us to make a direct comparison. These results differ from the data obtained in ovarian tissues (Scambia *et al.*, 1996), thus indicating the possibility that H2 may play a more important role in endometrium and cervix than in other tissues of the female genital tract. H2 has been reported as a very abundant message in breast cancer as well (Tokunaga *et al.*, 1993). A clear direct correlation was observed between the levels of expression of H1 and H2. A direct correlation has been reported earlier in ovarian and colon cancer (Mandai *et al.*, 1994; Myeroff and Markowitz, 1993; Scambia *et al.*, 1996), but has not been observed in other tissues. In breast cancer, for example, H1 and H2 levels are not correlated with each other and only H1 is related to prognosis (Tokunaga *et al.*, 1993). This suggests that different regulatory pathways, which control the absolute and relative levels of H1 and H2, may act in different tissues.

At present the clinical significance of nm23 in different malignancies has been poorly investigated. As far as gynaecological tumours are concerned, we have demonstrated that nm23-H1 expression has an independent favourable prognostic role in ovarian cancer (Scambia *et al.*, 1996). The possibility that nm23 expression may have a favourable significance in uterine tumours is also supported by the data reported here. In our series, patients with high levels of nm23 showed a lower incidence of lymph node involvement as had already been reported in breast (Bevilacqua *et al.*, 1989) and ovarian cancer (Viel *et al.*, 1995). Moreover, a high level of nm23 is also correlated with a lesser degree of myometrial invasion.

In conclusion, our data suggest that nm23 may play a role in the biology of endometrial and cervical cancer and support the undertaking of more extensive studies to evaluate whether nm23 may be considered an important marker for defining aggressiveness/metastatic potential of uterine tumours.

**Acknowledgements**
We thank Dr Patricia Steeg for providing the nm23-H1 and nm23-H2 probes and the anti-Nm23 polyclonal antibody. This work was partially supported by the Italian Association for Cancer Research (AIRC).

### References

AMBROS RA, VIGNA PA, FIGGE H, KALLAKURY BV, MASTRANGELO A, EASTMAN AY, MALFETANO J AND ROSS JS. (1994). Observations on tumor and metastatic suppressor gene status in endometrial carcinoma with particular emphasis on p53. *Cancer*, 73, 1686–1692.

AUSUBEL FM, BRENT R, KINGSTON RE, MOORE DD, SEIDMAN JG, SMITH JA AND STRUHL K. (1994). *Current Protocols in Molecular Biology*. John Wiley & Sons: Boston.

BENEDETTI-PANICI P, SCAMBIA G, BAIOCCHI G, GREGGI S, RAGUSA G, GALLO A, CONTE M, BATTAGLIA F, LAURELLI G, RABITTI C, CAPELLI A AND MANCUSO S (1991). Neoadjuvant chemotherapy and radical surgery in locally advanced cervical cancer. *Cancer*, **67**, 372–379.

BEVILACQUA G, SOBEL ME, LIOTTA LA AND STEEG PS. (1989). Association of low nm23 RNA levels in human primary infiltrating ductal breast carcinomas with lymph-node involvement and other histopathological indicators of high metastatic potential. *Cancer Res.*, **49**, 5185–5190.

CHOMCZYNSKI P AND SACCHI N. (1987). Single step method of RNA isolation by guanidium-thiocyanate-phenol-chloroform extraction. *Anal. Biochem.*, **162**, 156–159.

FLORENES VA, AAMDAL S, MYKLEBOST O, MAELANSMO GM, BRULAND DS AND FODSTAD D. (1992). Levels of nm23 messenger RNA in metastatic malignant melanomas: inverse correlation to disease progression. *Cancer Res.*, **52**, 6088–6091.

GILLES AM, PRESECAN E, VONICA AND LASCU I. (1991). Nucleoside diphosphate kinase from human erythrocytes. Structural characterization of the two polypeptide chains responsible for heterogeneity of hexameric enzyme. *J. Biol. Chem.*, **266**, 8784–8789.

HOWLETT AR, PETERSEN OW, STEEG PS AND BISSEL MJ. (1994). A novel function for the nm23-H1 gene: overexpression in human breast carcinoma cells leads to the formation of basement membrane and growth arrest. *J. Natl Cancer Inst.*, **86(24)**, 1838–1844.

JIANG XP, LIU KD AND ZHOU XD. (1994). Cloning and sequencing of nm23-H3b, a new gene identified to nm23. *Chinese Med. J.*, **74(11)**, 670–672.

KNECHT DA AND DIMOND RL. (1984). Visualization of antigenic proteins on Western blots. *Anal. Biochem.*, **136**, 180–184.

KONISHI N, NAKAOKA S, TSUZUKI T, MATSUMOTO K, KITAHORI Y, HIASA Y, URANO T AND SHIKU H. (1993). Expression of nm23-H1 and nm23-H2 proteins in prostate carcinoma. *Jpn J. Cancer Res.*, **84**, 1050–1054.

LEONE A, FLATOW U, KING CR, SANDEEN MA, MARGUILES IN, LIOTTA LA AND STEEG PS. (1991). Reduced tumor incidence, metastatic potential, and cytokine responsiveness of nm23-transfected melanoma cells. *Cell*, **65**, 25–39.

LEONE A, FLATOW U, VANHOUTTE K AND STEEG PS. (1993*a*). Transfection of human nm23-H1 into the human MDA-MB-435 breast carcinoma cell line: effects on tumor metastatic potential, colonization and enzymatic activity. *Oncogene*, **8**, 2325–2333.

LEONE A, SEEGER RC, HONG CM, ARBOLEDA MJ, BRODEUR GM, STRAM D, SLAMON DJ AND STEEG PS. (1993*b*). Evidence for nm23 RNA overexpression, DNA amplification and mutation in aggressive childhood neuroblastomas. *Oncogene*, **8**, 855–865.

LIOTTA LA AND STEEG PS. (1990). Clues to the function of nm23 and Awd proteins in development, signal transduction, and tumor metastasis provided by studies of Dictyostelium discoideum. *J. Natl Cancer Inst.*, **82**, 1170–1172.

LOMBARDI D, SACCHI A, D'AGOSTINO G AND TIBURSI G. (1995). The association of the nm23-M1 protein and beta-tubulin correlates with cell differentiation. *Exp. Cell Res.*, **217**, 267–271.

MACDONALD NJ, DE LA ROSA A, BENEDICT MA, FREIJE JM, KRUTSCH H AND STEEG PS. (1993). A serine phosphorilation of nm23 and not its nucleoside diphosphate kinase activity, correlates with suppression of tumor metastatic potential. *J. Biol. Chem.*, **268**, 25780–25789.

MANDAI M, KONISHI I, KOSHIYAMA M, MORI T, ARAO S, TASHIRO H, OKAMURA H, NOMURA H, HIAI H AND FUKUMOTO M. (1994). Expression and metastasis-related nm23-H1 and nm23-H2 genes in ovarian carcinomas: correlation with clinicopathology, EGFR, c-*erb*B-2 and c-*erb*B-3 genes, and sex steroid receptor expression. *Cancer Res.*, **54**, 1825–1830.

MYEROFF LL AND MARKOWITZ SD. (1993). Increased nm23-H1 and nm23-H2 messenger RNA expression and absence of mutations in colon carcinomas of low and high metastatic potential. *J. Natl Cancer Inst.*, **85**, 47–52.

NAKAYAMA T, OHTSURU A, NAKAO K, SHIMA M, NAKATA K, WATANABE K, ISHII N, KIMURA N AND NAGATAKI S. (1992). Expression in human hepatocellular carcinoma of nucleoside diphosphate kinase, a homologue of the nm23 gene product. *J. Natl Cancer Inst.*, **84**, 1349–1354.

NAKAYAMA H, YASUI W, YOKOZAKI H AND TOHARA E. (1993). Reduced expression of nm23 is associated with metastasis of human gastric carcinomas. *Jpn. J. Cancer Res.*, **84**, 184–190.

NICKERSON JA AND WELLS WW. (1984). The microtubule-associated nucleoside diphosphate kinase. *J. Biol. Chem.*, **259**, 1297–1304.

OHTSUKI K, IKEUCHI T AND YOKOYAMA M. (1986). Characterization of nucleoside diphosphate kinase-associated guanine nucleotide binding proteins from HeLa S3 cells. *Biochim. Biophys. Acta*, **882**, 322–330.

OKABE-KADO J, KASUKABE T, HONMA Y, HAYASHI M, HENZEL WJ AND HOZUMI M. (1992). Identity of a differentiation inhibiting factor for mouse myeloid leukemia cells with nm23/nucleotide diphosphate kinase. *Biochem. Biophys. Res. Commun.*, **182**, 987–994.

PETTERSON F. (1988). *Annual Report on the Results of Treatment in Gynecologic Cancer*. FIGO: Stockholm.

POSTEL EH AND FERRONE CA. (1994). Nucleoside diphosphate kinase enzyme activity of nm23-H2/PuF is not required for its DNA binding and *in vitro* transcriptional functions. *J. Biol. Chem.*, **269**, 8627–8630.

POSTEL EH, BERBERICH SJ, FLINT SJ AND FERRONE CA. (1993). Human c-myc transcription factor PuF identified as nm23-H2 nucleoside diphosphate kinase, a candidate suppressor of tumor metastasis. *Science*, **261**, 478–480.

SAMBROOK J, FRITSCH EF AND MANIATIS T. (1989). *Molecular Cloning: a Laboratory Manual*. 2nd edition. Cold Spring Harbor Laboratory Press: New York.

SCAMBIA G, CATOZZI L, BENEDETTI-PANICI P, FERRANDINA G, BATTAGLIA F, GIOVANNINI G, DISTEFANO M, PELLIZZOLA D, PIFFANELLI A AND MANCUSO S. (1993). Expression of ras p21 oncoprotein in normal and neoplastic human endometrium. *Gynecol. Oncol.*, **50**, 339–346.

SCAMBIA G, FERRANDINA G, MARONE M, BENEDETTI-PANICI P, GIANNITELLI C, PIANTELLI M, LEONE A AND MANCUSO S. (1996). Nm23 in ovarian cancer: correlation with clinical outcome and other clinico-pathological and biochemical prognostic parameters. *J. Clin. Oncol.*, **14**, 334–342.

STAHL JA, LEONE A, ROSENGARD AM, PORTER L, KING CR AND STEEG PS. (1991). Identification of a second human nm23 gene, nm23-H2. *Cancer Res.*, **51**, 445–449.

STEEG PS, BEVILACQUA G, KOPPER L, THORGEIRSSON UP, TALMADGE JE, LIOTTA LA AND SOBEL ME. (1988). Evidence for a novel gene associated with a low tumor metastatic potential. *J. Natl Cancer Inst.*, **80**, 200–204.

TOKUNAGA Y, URANO T, FURUKAWA K, KONDO H, KANEMATSU T AND SHIKU H. (1993). Reduced expression of nm23-H1, but not of nm23-H2, is concordant with the frequency of lymph-node metastasis of human breast cancer. *Int. J. Cancer*, **55**, 66–71.

VIEL A, DALL'AGNESE L, CANZONIERI V, SOPRACORDEVOLE F, CAPOZZI E, CARBONE A, VISENTIN MC AND BOIOCCHI M. (1995). Suppressive role of the metastasis-related nm23-H1 gene in human ovarian carcinomas: association of high messenger RNA expression with lack of lymph node metastasis. *Cancer Res.*, **55**, 2645–2650.

WALTON G AND GILL G. (1975). Nucleotide regulation of an eukaryotic protein synthesis initiation complex. *Biochim. Biophys. Acta*, **300**, 231–245.

WORLD HEALTH ORGANIZATION. (1979). *WHO Handbook for Reporting Results of Cancer Treatment*, no. 48. WHO: pp. 16–21. Geneva.